# Mobile Anchors

**DOI:** 10.1093/function/zqac024

**Published:** 2022-05-05

**Authors:** Antonio Zaza

**Affiliations:** Dipartimento di Biotecnologie e Bioscienze, Università degli Studi Milano-Bicocca, P.za della Scienza 2, 2016 Milano, Italy

**Keywords:** β-adrenergic receptors, AKAPs, nanodomains, signaling

## A Perspective on “Subcellular Propagation of Cardiomyocyte β-Adrenergic Activation of Calcium Uptake Involves Internal β-Receptors and AKAP7”

The least one expects from an anchor is to stay put (freely adapted from Salvador Dali's opinion about Alexander Calder's sculptures). The article by Shannon and co-workers in this issue^1^ contradicts such expectation, thus contributing an original and crucial tile to the puzzle of “nanodomain” signaling by β-adrenergic receptors (β-AR).

**Figure 1.**
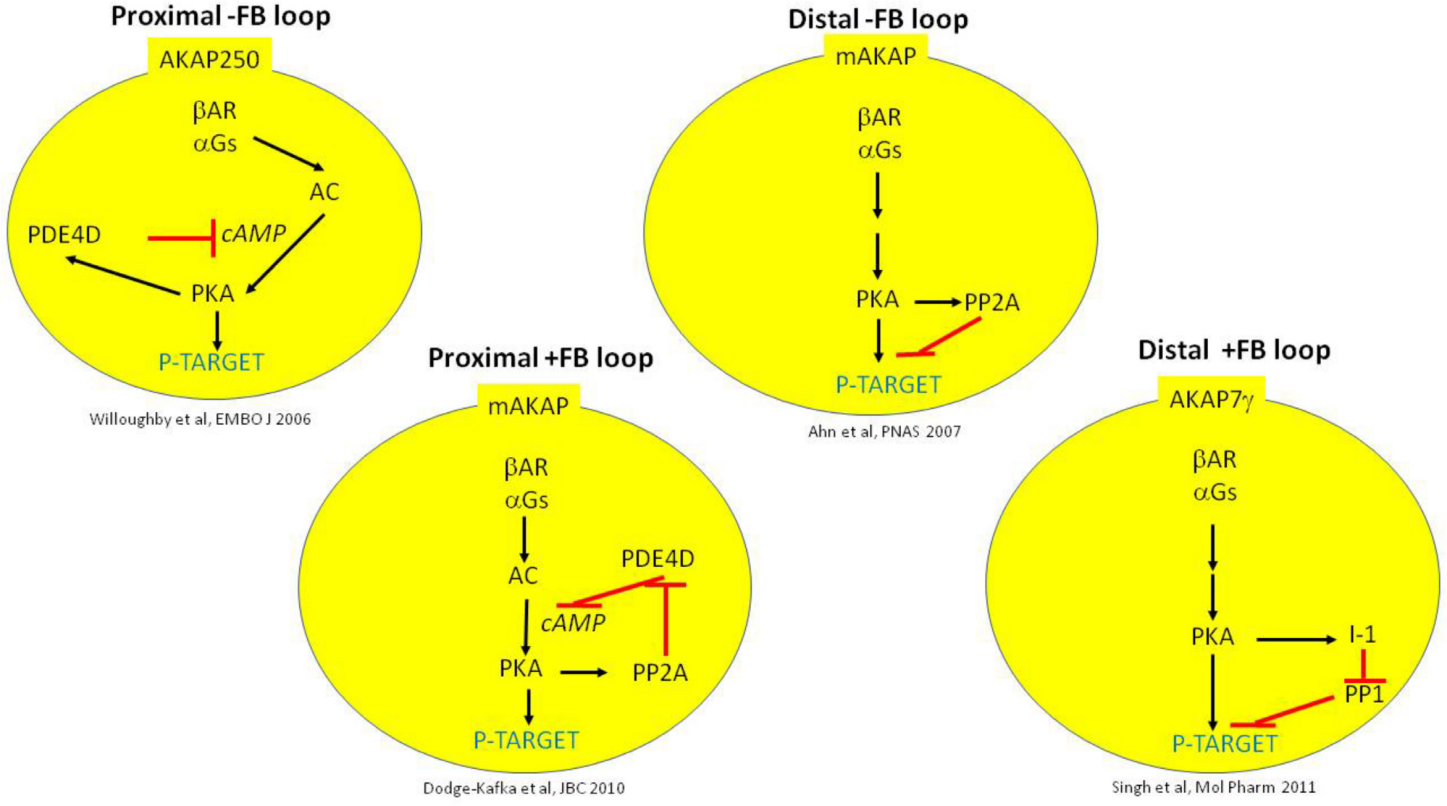
Examples of positive and negative feed-back loops organized within AKAP complexes. AC: adenylate cyclase; PKA: protein kinase A; PDE4D: prevailing cardiac phosphodiesterase; PP1 and PP2A: protein phosphatases; and I-1 inhibitor of PP1. References are quoted in full in the text or referred to in the quoted articles.

Mostly with the advent of dynamic FRET-based methodologies, the original concept of β-AR activating intracellular signals with diffuse intracellular targets has evolved to that of a system of confined receptor–signal complexes (nanodomains), each modulating functional elements according to their subcellular localization.^2^ This architecture allows to broaden considerably the spectrum of biological responses to a single extracellular agonist, still relying on a limited toolkit (the “usual suspects”) of receptor subtypes and downstream signals. This magic has two main ingredients: (1) the receptor, the downstream signals, and the target are in close proximity within the nanodomain,^3^ thus fostering mutual interactions; (2) the mutual interactions set up negative feed-back loops, which constrain signal duration and diffusion, or positive ones, which boost local signaling (Figure 1).^2^,^4–6^ Particularly for β2-AR, such an arrangement results in signals of limited duration (eg, minutes)^7^ and confined in space that is highly specific for a given subcellular domain. Disruption of membrane microdomains (eg, caveolae), as in hypertrophic remodeling, may result in loss of such specificity.^8^

“Kinase Anchoring Proteins,” AKAPs in the case of PKA signaling, are pivotal in clustering the receptor and its signaling molecules within a nanodomain.^2^ The multiplicity of AKAP isoforms allows to target the associated signaling complex to a specific PKA target. AKAPs disruption results in loss of signal confinement, possibly contributing to diffusion of sarcolemmal cAMP to deep targets (eg, phospholamban and sarcomeres) upon internalization of sarcolemmal β-AR.^2^

While such a “local control” architecture provides clear advantages in terms of versatility and resolution, it detracts from signal “amplification” (one agonist molecule acting on multiple receptors each activating multiple diffusible messengers, and so on) and “pleiotropy” (each messenger modulating multiple targets in a functionally concerted way), among the fundamental properties of receptor signaling. These properties have experimental support and would be structure-saving, because they reduce the number of elements needed to modulate global cell function. How can they be reconciled with the steadily emerging evidence of local control?

Local control and signaling pleiotropy, including deep targets, can be reconciled by the existence of complete intracellular receptor nanodomains activated by intracellular agonist, whose access to the cytosolic compartment is granted either by intrinsic liposolubility, or by specific sarcolemmal transporters (OCT for norepinephrine).^9^ An intracellular location has been reported for multiple receptors previously considered “sarcolemmal”; for these receptors, intracellular diffusion of the agonist (possibly hindered by its enzymatic degradation) may account for modulation of deep targets, with AKAPs granting preservation of local control. However, unlike signal amplification by freely diffusible molecules, local control implies a stiff relationship between the signaling complex and its target. Therefore, a disproportion between abundance of PKA and of the target to be phosphorylated can be hardly accommodated. This is the case of PKA phosphorylation of phospholamban (PLN), the latter being about 1000-fold more abundant than PKA.^10^

Shannon and coworkers^1^ address this issue by combining a localized perfusion strategy and FRET-based technology to β1-AR signaling in native rabbit ventricular myocytes. They show that the PLN-targeted intracellular β1AR-PKA-AKAP7γ complex they recently identified^9^ is endowed with considerable mobility, exceeding that of PLN itself. This implies that each activated PKA unit jumps from one PLN to the next, hence accomplishing signal amplification as required by the unbalanced stoichiometry. This process involves cyclic binding–unbinding of the signaling complex from PLN, made possible by the loss of PLN affinity, once phosphorylated, for AKAP7γ. Notably, the signaling complex may “surf” on the sarcoplasmic-reticulum (SR) membrane, possibly because AKAP7γ dimerization limits its 3D diffusion. In mobility (FRAP) experiments, AKAP7γ and PLN only were tagged; therefore, there was no direct evidence concerning the other components of the β-AR-PKA signaling cascade. Nonetheless AKAP7γ mobility was accompanied by diffusion, from the local agonist perfusion site, of the functional effects of phosphorylation. Persistence of norepinephrine (NE) effects in the presence of sotalol, a membrane impermeant β-AR antagonist, lends substantial support to the view that intracellular β-ARs contribute to PLN modulation. Interestingly, under sotalol, NE effect linearly increased throughout the 90 s recording; this was in sharp contrast with the rapidly saturating (within 50 s) response observed in the absence of sotalol.^1^ Thus, distinct signaling modalities, one recruiting sarcolemmal and intracellular β-ARs and the other based on intracellular ones only, modulate PLN with remarkably different kinetics. In terms of mechanism, short-range modulation of the β-AR signal within the AKAP nanodomain (Figure 1) might possibly account for signaling kinetics unlike those expected from a “bulk” signal activation—inactivation sequence. Whether such peculiarity of the intracellular signaling component may have a role in adrenergic modulation of SR function is unknown, and of potential significance in predicting specificities in the effect of AR agonists/antagonists according to their ability to enter the cell.

While AKAP7γ mobility is only one among the factors potentially contributing to spread β1-AR signaling cell wide, its description is crucial in reconciling the “local control” strategy with the signal amplification required for modulation of abundant targets. Albeit likely insufficient on its own, a further element to fill the stoichiometry gap might reside in PLN equilibrium between monomeric (SERCA inhibitory) and pentameric (noninhibitory) forms, the latter stabilized by PKA-mediated phosphorylation. By reducing availability of the monomeric form, pentamers phosphorylation contributes to SERCA stimulation by PKA^11^; thus, targeting PLN pentamers would amplify, theoretically by a factor of 5, the function of the AKAP7γ complex, irrespective of its mobility. Nonetheless, from Shannon and coworkers we learn that anchors’ mobility, far from being an oxymoron, may serve an important functional role.

The unexpected information emerging from detailed analysis of nanodomain signaling with advanced optical methodologies contributes to explain puzzling aspects of cell biology and pharmacology, identifies potential therapeutic targets, and may help in devising disease-specific strategies in the use of receptor antagonists.
